# Eating quickly is associated with waist-to-height ratio among Japanese adolescents: a cross-sectional survey

**DOI:** 10.1186/s13690-016-0130-3

**Published:** 2016-05-09

**Authors:** Hirotaka Ochiai, Takako Shirasawa, Hinako Nanri, Rimei Nishimura, Masaaki Matoba, Hiromi Hoshino, Akatsuki Kokaze

**Affiliations:** Department of Public Health, Showa University School of Medicine, 1-5-8 Hatanodai, Shinagawa-ku, Tokyo, 142-8555 Japan; Division of Diabetes, Metabolism and Endocrinology, Department of Internal Medicine, Jikei University School of Medicine, 3-25-8 Nishi-Shinbashi Minato-ku, Tokyo, 105-8461 Japan

**Keywords:** Eating quickly, Waist-to-height ratio, Adolescents, Eating until full

## Abstract

**Background:**

Central obesity, based on waist circumference (WC), has more adverse effects on health than general obesity, determined by body mass index. To date, eating quickly has been reported to be risk factors for overweight/obesity among children, adolescents, and adults. In contrast, there are few studies on the relationship between fast eating and central obesity among adolescents, particularly in Japan, where WC is not commonly measured in junior high schools. The aim of the present study was to investigate the relationship between eating quickly and waist-to-height ratio (WHtR), an index of central obesity, among adolescents in Japan.

**Methods:**

Study subjects were 2136 seventh-grade school children (12 or 13 years of age) from Ina town junior high schools in Japan, between 2004 and 2009. Measurements of height, weight, and WC were performed, and information about eating habits was collected using a self-administered questionnaire. A logistic regression model was used to calculate the odds ratio (OR) and 95 % confidence interval (95 % CI) for WHtR ≥ 0.5.

**Results:**

Eating quickly significantly increased the OR for WHtR ≥ 0.5 in boys (OR: 2.05, 95 % CI: 1.31–3.23) and girls (2.09, 1.15–3.81). When compared with the “not eating quickly and not eating until full” group, the OR for WHtR ≥ 0.5 in the “eating quickly and eating until full” group was 2.67 (95 % CI: 1.50–4.73) in boys and 2.59 (1.17–5.73) in girls, whereas that in the “eating quickly and not eating until full” group or the “not eating quickly and eating until full” group was not statistically significant regardless of sex.

**Conclusions:**

The present study showed that eating quickly was associated with WHtR ≥ 0.5, and “eating quickly and eating until full” had a substantial impact on WHtR ≥ 0.5 among adolescents. This study suggests that modifying fast eating to a slower pace may help prevent central obesity among adolescents.

## Background

Childhood obesity leads to many acute health problems and increases the risk of adult obesity, which in turn increases the likelihood of comorbidities [1]. The risk of obesity persisting into adulthood was shown to be higher among obese adolescents than among younger children [2]. Moreover, a recent study reported that metabolic and physiologic abnormalities associated with obesity in adolescence (e.g., hypertension, dyslipidemias, orthopedic problems, and type 2 diabetes) tend to track into adulthood along with the condition of obesity itself [3]. Thus, obesity in adolescence is a serious public health issue.

A previous study showed that waist circumference (WC) is more closely linked to cardiovascular disease risk factors than body mass index (BMI) [4]. It was recently reported that obesity-related health risk is explained by WC and not by BMI [5]. Furthermore, a systematic review showed that WC was a significant predictor of cardiometabolic outcomes more often than BMI [6]. In addition, the increased mortality risk related to excess body fat is reported to be mainly due to abdominal adiposity [7]. Therefore, central obesity, which is based on WC, is thought to be more pathogenic than general obesity as measured by BMI; for example, central obesity was indicated to be more important as a predictor of diabetes, hypertension, and cardiovascular disease than general obesity [8]. These studies suggest the importance of the prevention of central obesity.

Eating quickly has been reported to be a risk factor for overweight/obesity among children, adolescents, and adults [9–14]. Recent systematic reviews reported that eating quickly is associated with excess body weight [15, 16], although Leong et al. suggested that once women have reached mid-life, faster eating does not predict further weight gain [17]. Moreover, eating speed was reported to be significantly correlated with WC among adults [18]. In contrast, there are a limited number of studies about the relationship between fast eating and central obesity, especially among adolescents. If eating quickly is associated with central obesity, it may be possible to help prevent central obesity by modifying the habit of eating quickly. Thus, it is important to investigate the relationship between eating quickly and central obesity among adolescents, particularly in Japan, where WC is not commonly measured in junior high schools.

In addition to eating quickly, eating until full, which refers to eating a large quantity of food in one meal and is unrelated to eating disorders, was reported to be associated with overweight/obesity [10]. Furthermore, a recent study showed that the impact of eating quickly and eating until full on overweight/obesity was stronger than that of eating quickly and not eating until full [12]. Therefore, it is necessary to consider eating until full when examining the relationship between eating quickly and central obesity because the influence of fast eating on central obesity could differ by eating until full or not. We hypothesized that “eating quickly and eating until full” may be associated with central obesity. Based on previous studies [19, 20], waist-to-height ratio (WHtR) was used as an index of central obesity in this study.

Accordingly, the aim of the present study was to investigate the relationship between eating quickly and WHtR and then to examine the impact of eating habit patterns (not eating quickly and not eating until full, eating quickly and not eating until full, not eating quickly and eating until full, eating quickly and eating until full) on WHtR among adolescents in Japan.

## Methods

### Study participants

Study subjects were all 2136 seventh-grade school children (12 or 13 years of age) from three Ina town junior high schools between 2004 and 2009. Ina-town in Saitama Prefecture, Japan, had implemented a unique health check-up program as a part of its community health services. In the program, a questionnaire survey and anthropometric measurements were performed [21, 22]. Written informed consent was obtained from the parent or guardian of each subject prior to the subject’s participation in this study. The study protocol was approved by the Medical Ethics Committee of Showa University School of Medicine (Approval No. 127).

Among all 2136 subjects, 26 refused to participate in the program (participation rate: 98.8 %), and 182 were excluded due to missing data about variables in this study. Thus, data from 1928 participants were analyzed.

### Questionnaire survey

The following information was collected using a self-administered questionnaire for each child: sex, age, exercise other than physical education class (daily, sometimes, or none), snack after dinner (always, often, seldom, or none), eating speed, and eating until full. Information about eating speed was obtained from three qualitative categories (fast, medium, or slow) [23] to the question “How fast is your eating speed compared to others?” With regard to eating until full, responses were given as a yes or no [24].

In addition, the parent or guardian of each participant was asked to fill in a self-administered questionnaire about the participant’s frequency of eating breakfast (daily, sometimes, or none). Frequency of eating breakfast was categorized into the following two groups: skipping breakfast (sometimes and none) and not skipping breakfast (daily).

### Anthropometric measurements

The measurements of each participant’s height, weight, and WC were performed by trained school nurses/doctors either in the school’s infirmary or in a designated room to protect the privacy of participants. Before the measurements, the calibration was done. For anthropometric measurements, participants were lightly clothed and were barefoot. Height and weight of each participant were measured to the nearest 0.1 cm using a stadiometer and to the nearest 0.1 kg using a scale. BMI was calculated as weight (kg)/height^2^ (m^2^). WC was measured to the nearest 0.1 cm in a standing position at the navel level while another examiner checked verticality from the side. WHtR was calculated as WC (cm)/height (cm). These measurements were recorded annually from 2004 to 2009. The same measurement protocol was used annually throughout the study period.

### Data analysis

Statistical analysis was performed separately for each sex. Data were presented as median (25, 75th percentile) for continuous variables or number (%) for categorical variables. In accordance with previous studies [10, 23, 25], eating speed was categorized into two groups in the analysis: eating quickly (fast) or not eating quickly (medium or slow). The Wilcoxon rank-sum test, the chi-square test, or Fisher’s exact test was used to compare various characteristics between the WHtR ≥ 0.5 group and the WHtR < 0.5 group, which were determined by previous studies [19, 20, 26, 27]. A logistic regression model was used to calculate the odds ratio (OR) for WHtR ≥ 0.5 and the 95 % confidence interval (95 % CI). A *P* value < 0.05 was considered statistically significant. Statistical analyses were performed using Statistical Analysis System software (version 9.4; SAS Institute Inc., Cary, NC, USA).

## Results

Characteristics of the WHtR ≥ 0.5 and the WHtR < 0.5 groups in boys (*n* = 970) are shown in Table 1. Anthropometric variables in the WHtR ≥ 0.5 group were higher than those in the WHtR < 0.5 group. There was a statistically significant difference between the WHtR ≥ 0.5 and the WHtR < 0.5 groups in exercise. The proportion of those who ate quickly in the WHtR ≥ 0.5 group was significantly higher than that in the WHtR < 0.5 group.

**Table 1:** Characteristics of boys by waist-to-height ratio

Variables	Waist-to-height ratio		*P*-value ^a^
	<0.5 (*n* =878)	≥0.5 (*n* =92)	
Age (years)	12.0 (12.0, 13.0)	12.0 (12.0, 13.0)	0.165
Height (cm)	154.7 (148.4, 160.0)	156.1 (151.3, 161.4)	0.063
Weight (kg)	42.5 (37.4, 47.8)	60.3 (55.1, 70.0)	<0.001
Body mass index (kg/m^2^)	17.7 (16.5, 19.1)	25.1 (23.1, 27.4)	<0.001
Waist circumference (cm)	62.6 (59.5, 66.3)	84.9 (79.6, 91.9)	<0.001
Waist-to-height ratio	0.40 (0.39, 0.43)	0.54 (0.51, 0.58)	<0.001
Exercise, *n* (%)			
Daily	759 (86.5)	67 (72.8)	0.001
Sometimes	58 (6.6)	10 (10.9)	
None	61 (7.0)	15 (16.3)	
Skipping breakfast, *n* (%)			
Yes	41 (4.7)	3 (3.3)	0.791
No	837 (95.3)	89 (96.7)	
Snack after dinner, *n* (%)			
Always or sometimes	486 (55.4)	3 (3.3)	0.434
Seldom or none	392 (44.7)	89 (96.7)	
Eating quickly, *n* (%)			
Yes	218 (24.8)	37 (40.2)	0.001
No	660 (75.2)	55 (59.8)	
Eating until full, *n* (%)			
Yes	461 (52.5)	54 (58.7)	0.258
No	417 (47.5)	38 (41.3)	

Except where indicated *n* (%), values are median (25, 75th percentile)

^a^ Wilcoxon rank-sum test, chi-squared test, or Fisher's exact test

Table 2 shows characteristics of the WHtR ≥ 0.5 and the WHtR < 0.5 groups in girls (*n* = 958). Anthropometric variables were higher in the WHtR ≥ 0.5 group than in the WHtR < 0.5 group. A statistically significant difference was found between the WHtR ≥ 0.5 and the WHtR < 0.5 groups in exercise. A significantly higher proportion of those who ate quickly was observed in the WHtR ≥ 0.5 group compared with the WHtR < 0.5 group.

**Table 2:** Characteristics of girls by waist-to-height ratio

Variables	Waist-to-height ratio		*P*-value ^a^
	<0.5 (*n* =886)	≥0.5 (*n* =72)	
Age (years)	12.0 (12.0, 13.0)	12.0 (12.0, 13.0)	0.027
Height (cm)	152.7 (148.9, 156.8)	153.7 (149.8, 159.1)	0.093
Weight (kg)	42.4 (38.1, 46.7)	57.9 (53.1, 62.3)	<0.001
Body mass index (kg/m^2^)	18.1 (16.7, 19.5)	24.3 (22.9, 25.7)	<0.001
Waist circumference (cm)	63.5 (60.0, 67.0)	80.9 (77.5, 84.1)	<0.001
Waist-to-height ratio	0.42 (0.40, 0.44)	0.52 (0.51, 0.55)	<0.001
Exercise, *n* (%)			
Daily	545 (61.5)	29 (40.3)	0.001
Sometimes	104 (11.7)	16 (22.2)	
None	237 (26.8)	27 (37.5)	
Skipping breakfast, *n* (%)			
Yes	53 (6.0)	7 (9.7)	0.205
No	833 (94.0)	65 (90.3)	
Snack after dinner, *n* (%)			
Always or sometimes	469 (52.9)	34 (47.2)	0.351
Seldom or none	417 (47.1)	38 (52.8)	
Eating quickly, *n* (%)			
Yes	110 (12.4)	16 (22.2)	0.018
No	776 (87.6)	56 (77.8)	
Eating until full, *n* (%)			
Yes	477 (53.8)	40 (55.6)	0.779
No	409 (46.2)	32 (44.4)	

Except where indicated *n* (%), values are median (25, 75th percentile)

^a^ Wilcoxon rank-sum test, chi-squared test, or Fisher's exact test

The crude and adjusted ORs of eating quickly or eating until full for WHtR ≥ 0.5 were calculated (Table 3). Eating quickly significantly increased the OR for WHtR ≥ 0.5 in boys (OR: 2.05, 95 % CI: 1.31–3.23). A significantly increased OR of eating quickly was also found among girls (2.09, 1.15–3.81). The OR of eating until full was not statistically significant. Additionally, BMI and WC were significantly higher in boys who ate quickly (median, BMI: 18.9 kg/m^2^ and WC: 65.7 cm) than in those who didn’t eat quickly (17.6 kg/m^2^ and 62.5 cm), whereas no statistically significant differences were observed between those who ate until full (17.9 kg/m^2^ and 63.2 cm) and those who didn’t eat until full (18.0 kg/m^2^ and 63.2 cm). In girls, BMI and WC were significantly higher in those who ate quickly (median, BMI: 19.2 kg/m^2^ and WC: 66.1 cm) than in those who didn’t eat quickly (18.1 kg/m^2^ and 63.8 cm), whereas there were no statistically significant differences between those who ate until full (18.3 kg/m^2^ and 64.0 cm) and those who didn’t eat until full (18.3 kg/m^2^ and 64.0 cm).

**Table 3:** Crude and adjusted odds ratios of eating quickly or eating until full for waist-to-height ratio (WHtR) ≥ 0.5

Variables	Total	WHtR ≥ 0.5	Crude		Adjusted	
	N	*n* (%)	OR (95% CI)	*P*-value	OR (95% CI)	*P*-value
Boys						
Eating quickly						
Yes	255	37 (14.5)	2.04 (1.31–3.18)	0.002	2.05 (1.31–3.23)	0.002
No	715	55 (7.7)	1.00		1.00	
Eating until full						
Yes	515	54 (10.5)	1.29 (0.83–1.99)	0.259	1.25 (0.80–1.95)	0.321
No	455	38 (8.4)	1.00		1.00	
Girls						
Eating quickly						
Yes	126	16 (12.7)	2.02 (1.12–3.64)	0.020	2.09 (1.15–3.81)	0.016
No	832	56 (6.7)	1.00		1.00	
Eating until full						
Yes	517	40 (7.7)	1.07 (0.66–1.74)	0.779	1.12 (0.68–1.82)	0.662
No	441	32 (7.3)	1.00		1.00	

*OR*, odds ratio, *CI*, confidence interval

Adjusted for age, exercise, skipping breakfast, and snack after dinner

The crude and adjusted ORs for WHtR ≥ 0.5 were shown by eating habit patterns based on eating quickly and eating until full; “not eating quickly and not eating until full”, “eating quickly and not eating until full”, “not eating quickly and eating until full”, and “eating quickly and eating until full” (Table 4). In boys, “eating quickly and eating until full”significantly increased the OR (OR: 2.67, 95 % CI: 1.50–4.73) when compared with “not eating quickly and not eating until full”. In girls, “eating quickly and eating until full” showed a significantly increased OR for WHtR ≥ 0.5 (2.59, 1.17–5.73). There were statistically significant differences among eating habit patterns in BMI (median: 17.9 kg/m^2^ in the “not eating quickly and not eating until full” boys, 18.2 kg/m^2^ in the “eating quickly and not eating until full” boys, 17.3 kg/m^2^ in the “not eating quickly and eating until full” boys, and 19.5 kg/m^2^ in the “eating quickly and eating until full” boys) and in WC (62.8 cm, 64.6 cm, 62.0 cm, and 66.4 cm, respectively). In girls, statistically significant differences among eating habit patterns were found in BMI (median: 18.1 kg/m^2^ in the “not eating quickly and not eating until full” group, 19.0 kg/m^2^ in the “eating quickly and not eating until full” group, 18.1 kg/m^2^ in the “not eating quickly and eating until full” group, and 19.3 kg/m^2^ in the “eating quickly and eating until full” group) and in WC (63.9 cm, 66.7 cm, 63.6 cm, and 65.5 cm, respectively).

**Table 4:** Crude and adjusted odds ratios of eating habit patterns based on eating quickly and eating until full for waist-to-height ratio (WHtR) ≥ 0.5

Variables	Total	WHtR ≥ 0.5	Crude		Adjusted	
	N	*n* (%)	OR (95% CI)	*P*-value	OR (95% CI)	*P*-value
Boys						
Not eating quickly and	339	29 (8.6)	1.00		1.00	
not eating until full						
Eating quickly and	116	9 (7.8)	0.90 (0.41–1.96)	0.789	0.88 (0.40–1.94)	0.747
not eating until full						
Not eating quickly and	376	26 (6.9)	0.79 (0.46–1.38)	0.412	0.76 (0.44–1.33)	0.335
eating until full						
Eating quickly and	139	28 (20.1)	2.70 (1.54–4.74)	<0.001	2.67 (1.50–4.73)	<0.001
eating until full						
Girls						
Not eating quickly and	385	26 (6.8)	1.00		1.00	
not eating until full						
Eating quickly and	56	6 (10.7)	1.66 (0.65–4.22)	0.290	1.60 (0.62–4.14)	0.330
not eating until full						
Not eating quickly and	447	30 (6.7)	0.99 (0.58–1.71)	0.981	1.02 (0.59–1.76)	0.952
eating until full						
Eating quickly and	70	10 (14.3)	2.30 (1.06–5.02)	0.036	2.59 (1.17–5.73)	0.019
eating until full						

*OR*, odds ratio, *CI*, confidence interval

Adjusted for age, exercise, skipping breakfast, and snack after dinner

## Discussion

The present study investigated the relationship between eating quickly and WHtR among adolescents in Japan. Results showed that eating quickly was associated with WHtR ≥ 0.5 and “eating quickly and eating until full” had a substantial impact on WHtR ≥ 0.5 for each sex. To the best of our knowledge, this is the first study about the association between eating quickly and WHtR among adolescents in Japan.

In this study, eating quickly significantly increased the OR for WHtR ≥ 0.5 in each sex. A recent study showed that eating rapidly was associated with larger WC [28]. In addition, self-reported faster eating was shown to be positively associated with visceral fat accumulation [29]. Furthermore, some studies among adults have reported that eating fast was associated with metabolic syndrome [30, 31]. These study results support that the result of this study was reasonable.

When compared to the “not eating quickly and not eating until full” group, “eating quickly and eating until full” significantly increased the OR for WHtR ≥ 0.5, whereas no significantly increased ORs were observed in the “eating quickly and not eating until full” group in the present study. The reason could be due to the difference of the total energy intake between in the “eating quickly and eating until full” group and in the “eating quickly and not eating until full” group. Recent studies reported that eating rate affects energy intake [32], and energy intake per day increased significantly with the increase in the rate of eating [33]. In addition, Maruyama et al. showed that total energy intake in the “eating quickly and eating until full” group was higher than that in the “eating quickly and not eating until full” group [10]. Because information regarding total energy intake was not obtained in the present study, further study will be needed to verify our study results.

In our study, eating quickly was significantly associated with WHtR ≥ 0.5 and the significantly increased OR for WHtR ≥ 0.5 was observed only in the “eating quickly and eating until full” group among eating habit patterns regardless of sex. These results suggest that “eating quickly and eating until full” had a substantial impact on WHtR ≥ 0.5. A recent study showed that reducing eating rate may be an effective intervention to decrease energy intake [32]. In addition, Andrade et al. reported that eating slowly may help to maximize satiation and reduce energy intake within meals [34]. Moreover, it was recently shown that modification of eating rate could be an efficient, cost effective adjunct for promoting healthy eating and decreasing energy intake [35]. Therefore, modifying fast eating to slower pace could be effective for the decrease of energy intake, which contributes to the prevention of the central obesity among adolescents.

The strength of the present study is that the participation rate was over 95 %, which suggests that the effect of sampling bias on the present study results was small. Furthermore, the outcome of this study (WHtR) was defined by the measurements of height and WC among over 2000 adolescents; WC is not usually measured in annual health examinations at junior high schools in Japan. However, this study has some limitations. First, the results of this study might be affected by some potential confounders such as total energy intake, daily eating frequency, eating out, type of food, family educational level, parental WHtR, and parental smoking [9, 16, 26, 28, 30, 36]. Because these items were not obtained in our study, the influence of residual confounding on the present study results cannot be excluded. Second, the information about eating quickly and eating until full was self-reported. However, a recent study reported that self-reported eating rate aligned with laboratory measured eating rate [35]. Moreover, Otsuka et al. showed a statistically positive association between self-reported rate of eating and energy intake [33]. In addition, a previous study reported that total energy intake in participants who reported eating until full was higher than that in those who did not report eating until full [10]. These study results support that our study results were reasonable. Third, participants in our study were from one town in Japan, which might limit the ability to generalize our findings to other populations. Finally, our study design is cross-sectional. Thus, the possibility of reverse causality cannot be ruled out.

## Conclusions

The present study showed that eating quickly was associated with WHtR ≥ 0.5 among adolescents. Moreover, eating quickly and eating until full had a substantial impact on WHtR ≥ 0.5. This study suggests that modifying fast eating to a slower pace may help prevent central obesity among adolescents.

## Abbreviations

BMI: body mass index
CI: confidence interval
OR: odds ratio
WC: waist circumference
WHtR: waist-to-height ratio
